# Gluconeogenesis and glycogenolysis required in metastatic breast cancer cells

**DOI:** 10.3389/fonc.2024.1476459

**Published:** 2024-10-16

**Authors:** Emily Hicks, Marjorie Anne Layosa, Chaylen Andolino, Caitlin Truffer, Yazhen Song, Timothy D. Heden, Shawn S. Donkin, Dorothy Teegarden

**Affiliations:** ^1^ Department of Nutrition Science, Purdue University, West Lafayette, IN, United States; ^2^ Purdue Institute for Cancer Research, Purdue University, West Lafayette, IN, United States; ^3^ Pennington Biomedical Research Center, Baton Rouge, LA, United States; ^4^ Department of Animal Sciences, Purdue University, West Lafayette, IN, United States; ^5^ College of Agricultural Sciences, Oregon State University, Corvallis, OR, United States

**Keywords:** glucose, glycogen, gluconeogenesis, glycogenolysis, glycophagy, breast cancer

## Abstract

**Introduction:**

Metabolic adaptability, including glucose metabolism, enables cells to survive multiple stressful environments. Glycogen may serve as a critical storage depot to provide a source of glucose during times of metabolic demand during the metastatic cascade; therefore, understanding glycogen metabolism is critical. Our goal was to determine mechanisms driving glycogen accumulation and its role in metastatic (MCF10CA1a) compared to nonmetastatic (MCF10A-*ras*) human breast cancer cells.

**Methodology:**

^13^C_6_-glucose flux analysis in combination with inhibitors of the gluconeogenic pathway via phosphoenolpyruvate carboxykinase (PCK), the anaplerotic enzyme pyruvate carboxylase (PC), and the rate-limiting enzyme of the pentose phosphate pathway (PPP) glucose 6-phosphate dehydrogenase (G6PD). To determine the requirement of glycogenolysis for migration or survival in extracellular matrix (ECM) detached conditions, siRNA inhibition of glycogenolysis (liver glycogen phosphorylase, PYGL) or glycophagy (lysosomal enzyme α-acid glucosidase, GAA) enzymes was utilized.

**Results:**

Metastatic MCF10CA1a cells had 20-fold greater glycogen levels compared to non-metastatic MCF10A-*ras* cells. Most glucose incorporated into glycogen of the MCF10CA1a cells was in the five ^13^C-containing glucose (M+5) instead of the expected M+6 glycogen-derived glucose moiety, which occurs through direct glucose conversion to glycogen. Furthermore, ^13^C_6_-glucose in glycogen was quickly reduced (~50%) following removal of ^13^C-glucose. Incorporation of ^13^C_6_-glucose into the M+5 glucose in the glycogen stores was reduced by inhibition of PCK, with additional contributions from flux through the PPP. Further, inhibition of PC reduced total glycogen content. However, PCK inhibition increased total unlabeled glucose accumulation into glycogen, suggesting an alternative pathway to glycogen accumulation. Inhibition of the rate-limiting steps in glycogenolysis (PYGL) or glycophagy (GAA) demonstrated that both enzymes are necessary to support MCF10CA1a, but not MCF10A-*ras*, cell migration. GAA inhibition, but not PYGL, reduced viability of MCF10CA1a cells, but not MCF10A-*ras*, in ECM detached conditions.

**Conclusion:**

Our results indicate that increased glycogen accumulation is primarily mediated through the gluconeogenesis pathway and that glycogen utilization is required for both migration and ECM detached survival of metastatic MCF10CA1a cells. These results suggest that glycogen metabolism may play an important role in the progression of breast cancer metastasis.

## 1 Introduction

Breast cancer is a major public health concern as it is the second leading cause of cancer-related deaths (1). In particular, breast cancer metastasis is of particular concern as the five-year survival rate drops from 99% to only 30% if the cancer has metastasized at the time of diagnosis (1). The complex multi-step process of the metastatic cascade and successful colonization in a secondary site is proposed to require dysregulation of cancer cell energy metabolism, including glucose metabolism, where the metabolic reprogramming promotes survival and metastatic progression in adverse environments, such as migration or extracellular matrix (ECM) detached conditions (1–3).

In healthy physiological circumstances, glucose metabolism is a highly regulated process; however, the process is dysregulated in many cancer cells and supports key processes of the metastatic cascade (4, 5). Evidence suggests that many tumor cells increase glucose uptake, glycolysis, and lactate production (6, 7). Upregulated glycolysis is proposed to promote cellular proliferation by providing carbon backbones for essential components of growth (8). Additionally, in healthy conditions when extracellular glucose levels are low, gluconeogenesis, through phosphoenolpyruvate carboxykinase (PCK), which converts oxaloacetate to phosphoenolpyruvate (PEP), functions to produce needed glucose from pyruvate as well as from other non-carbohydrate sources that contribute to the TCA cycle. There are two PCK isoforms, the mitochondrial form (PCK2) and cytosolic form (PCK1), encoded by separate genes. PCK1 is highly enriched in gluconeogenic organs such as the liver whereas PCK2 is widely expressed and can be induced by diverse stress. In addition to glucose biosynthesis, PCK plays an important role in glycerol biosynthesis, serine biosynthesis, and amino acid metabolism (9). Evidence suggests that PCK plays a role in dysregulated glucose metabolism as it is upregulated in various cancers, driving the production of glucose which supports sustained tumor initiation, growth, and regulation of cancer stem cells (10–13).

Glycogen, a polysaccharide of linked glucose molecules that serves as a storage form of glucose, is produced through glycogenesis in healthy physiological circumstances. Glycogen is primarily stored in muscle and liver, although the brain and heart also contain minor stores (14). The highest levels of glycogen are accumulated in the muscle, where the enzyme to dephosphorylate and release glucose, glucose 6-phosphatase, is not expressed. On the other hand, hepatic expression and hormonal regulation of this enzyme allows cellular glycogen stores to support overall body glucose homeostasis (14). Glycogenesis is mediated by glycogen synthase (GYS) which is activated by glucose 6-phosphate, when glucose levels are elevated. GYS is downregulated through phosphorylation by the serine/threonine glycogen synthase kinase 3 (GSK3) (15).

Conversely, glycogenolysis to release glucose from glycogen stores as physiological needs arise utilizes both glycogenolysis (cytosolic) or glycophagy (lysosomal) (14). Cytosolic glycogen degradation is mediated by the rate-limiting enzymes glycogen debranching enzyme and glycogen phosphorylase (PYG). PYG is activated by phosphorylase kinase to release glucose 1-phosphate from glycogen, a process inhibited by phosphorylase kinase. Phosphorylation of this enzyme by protein kinase A (PKA, or cAMP-dependent protein kinase), however, reduces its inhibitory activities. Additionally, glycogen is targeted to lysosomes via the chaperone protein starch binding domain containing protein (STBD1), where it is cleaved through the process of glycophagy, which is mediated by acid α-glucosidase (GAA) in hepatocytes. Evidence suggests that in skeletal muscle glycogen delivery to lysosomes occurs independently of STBD1 (16). Thus, similar to glucose utilization, the process of glycogen synthesis and degradation is a highly regulated process, which in healthy physiological conditions is dependent on the cellular or overall body requirements for glucose.

Glucose release from accumulated glycogen may function as a reserve energy source to supply glucose in stressful conditions during metastasis (4). Support for this concept includes the evidence that various cancer types have greater stores of glycogen compared to benign cells (15, 17). In addition, the regulation of glycogen metabolism has been studied in breast, liver, lung, kidney, ovary, bladder, and blood cancers, where dysregulation of glycogen metabolism is linked to advanced metastatic disease (17). For example, genes involved in glycogen biosynthesis, including GYS, are upregulated and promote glycogen accumulation in response to elevated hypoxia-inducible factor-1α (HIF-1α) protein abundance, which is also associated with breast cancer aggressiveness (17–21). Further, the accumulation of glycogen within cancer cells is associated with the expression of oncogenes such as RAB25 (17), which upregulates proliferation and migration via its roles in endosomal cycling and regulation of cell signal transduction (22). The upregulation of RAB25 in ovarian carcinoma cells induces the increased binding of RNA Polymerase II-specific transcription factor TFIIB to HIF-1α at the promoter of the target gene, glucose transporter type 1 (GLUT1) (23), potentially promoting increased synthesis and accumulation of glycogen through increased glucose uptake (17, 24). PYG, providing glucose for utilization, also supports proliferation in some cancer types (25). Thus, previous literature suggests that glycogen accumulation and dysregulation of glycogenolysis in cancer may play a role in metastatic progression.

Despite the previous literature supporting a potential role, glycogen metabolism is poorly understood in the context of cancer progression. The goal of the current studies is to determine the cellular pathways utilized to accumulate glycogen in metastatic breast cancer cells, and to elucidate the role of glycogen utilization through glycolysis or glycophagy to support migration or survival in ECM detached conditions, two critical steps in the metastatic cascade.

## 2 Materials and methods

### 2.1 Cell culture

The MCF10A-*ras* human breast cancer cell line was derived from the non-malignant human breast MCF10A epithelial cell line by Harvey-*ras* oncogene transfection. The MCF10A-*ras* cells form primary tumors *in vivo*, but do not metastasize. The MCF10CA1a cells were derived from the MCF10A-*ras* cells and form primary tumors that metastasize to the lungs *in vivo* (26, 27). The MCF10A-*ras* (non-metastatic) and MCF10CA1a (metastatic) breast cancer cells were utilized in the experiments. Both cell types were cultured at 37°C with 5% CO_2_ in Dulbecco’s Modified Eagle Medium/Nutrient Mixture F-12 (DMEM/F-12, Sigma, St. Louis, MO) with 17.5 mM glucose and 2.5 mM glutamine. Complete MCF10A-*ras* cell culture media also contained 10 mg/L insulin (Sigma), 50 µg/L cholera toxin (Sigma), 20 µg/L epidermal growth factor (Sigma, St. Louis, MO), and 50 mg/L hydrocortisone (Sigma). In addition, media contained a final concentration of 1% penicillin/streptomycin antibiotic solution (Gibco) and 5% horse serum (Gibco, Waltham MA). All experiments were replicated at least twice.

### 2.2 Chemicals, reagents, and siRNA

cPEPCK GTP-competitive inhibitor (PEPCKi), which inhibits both PCK1 and PCK2, was from Axon Medchem (Reston, VA), and glucose 6-phosphate dehydrogenase inhibitor (G6PDi) and amylo-1,6-glucosidase were from Sigma (St. Louis, MO). The stable isotope ^13^C_6_-glucose was from Cambridge Isotope Laboratories, Inc (Tewksbury, MA). PYGL and GAA SMARTpool siRNAs and non-targeting siRNA (siCTRL) were acquired from Horizon Discovery (Waterbeach, UK) (**Table 1**).

**Table 1:** siRNA gene sequences.

siRNA	Target Sequences
**siPYGL**	GAAAGACCCUAAGAAGUUA GGAAGGAGCUAAGAUUGAA AGUUAUCAUUGGUGGUAAA UUGGAGAACUACAGAGUAU
**siGAA**	GAUCGUGAAUGAGCUGGUA GGAGGGACUUCACGUUCAA AGAGCGUGGUGCAGCAGUA GAAAUGGGCUACACGGCCA
**Non-targeting siRNA (siCTRL)**	UGGUUUACAUGUCGACUAA

### 2.3 ^14^C-glucose uptake

Cells were seeded into 60-mm dishes and grown for 48 hrs. After the incubation period, medium was changed and spiked with D-[^14^C(U)]-Glucose (0.25 µCi/mL) (PerkinElmer, Waltham, MA) then incubated at 37°C for 10 mins. Cells were washed twice, harvested with cold PBS, and added to a scintillation fluid. The cellular uptake of ^14^C-labeled were quantified using a Tri-Carb 5110TR 110 V Liquid Scintillation Counter (PerkinElmer, Waltham, MA). Another plate representative of plates used for substrate uptake measurement per cell line was incubated with 0.1N NaOH overnight and protein was quantified using bicinchoninic acid assay (BCA, ThermoFisher, Waltham, MA) protein kit. Radioactivity was expressed as the number of decompositions per min (dpm) normalized to protein.

### 2.4 Glycogen quantification assay

Cells were plated with complete media and grown to 80% confluence. Media was removed and cells were scraped into 10 mM sodium acetate buffer (pH 4.6). Amylo-1-6-glucosidase was added to cell lysate and incubated at 37°C for 2 hrs to hydrolyze glucose from glycogen. Samples were centrifuged (10,000 RPM) at room temperature and the supernatant analyzed utilizing the Glucose (GO) assay kit (Sigma) following the manufacturer’s protocol. Samples without amylo-1-6-glucosidase were processed similarly to the cell samples and subtracted from the results as a control for free cellular glucose. Cell pellets were analyzed for protein content (BCA assay) and results were normalized to protein. Data is expressed as µg glycogen/mg protein.

### 2.5 Glycogen synthesis

Cells were grown to 80% confluency and media replaced with 100% ^13^C_6_-glucose (17.5 mM) containing media 24 hrs prior to harvest. Media was removed and cells were scraped into 10 mM sodium acetate buffer (pH 4.6) and glycogen content assessed as described above and cell pellets were analyzed for protein content (BCA assay). The supernatant was dried utilizing a Speedvac (Thermo Savant SPD2010) and adonitrile-pentaacetate derivatization was completed on dried samples. Nitrilation was performed by adding hydroxylammonium chloride (0.2 mol/L, pyridine solution, Sigma) to the dried sample and heated at 90°C for 40 min. To acetylate the glucose, acetic anhydride (Sigma) was added, and samples heated at 90°C for 60 min. Samples were dried under nitrogen gas stream at 50°C and redissolved in ethyl acetate. The supernatant was analyzed utilizing gas chromatography mass spectroscopy (GC-MS) (Thermo TSQ 8000 triple quadrupole mass spectrometer coupled with a Thermo Trace 1310 gas chromatography). Data was collected through mass spectra analysis utilizing Chromeleon 7 software (ThermoFisher). Peaks for specific metabolites were selected utilizing standards and mean peak area was determined. Data is expressed as mean peak area normalized to protein and is expressed as mean peak area/µg protein.

### 2.6 Glycogenolysis

Similar to the methods described for glycogen synthesis, cells were labeled for 24 hrs in modified DMEM/F12 with 100% ^13^C_6_-glucose. After 24 hrs, media was removed and replaced with complete DMEM/F12 cell culture media without ^13^C_6_-glucose for an additional 3 hrs. Time 0 was harvested immediately after removal of ^13^C_6_-glucose media following the same procedure as above. After 3 hrs, samples were harvested, and cell pellets analyzed for protein content (BCA assay). The supernatant was dried and subjected to adonitrile-pentaacetate derivatization and analyzed utilizing GC/MS as described above.

### 2.7 RNA extraction and qRT-PCR

RNA was isolated from cell samples utilizing TRI-Reagent (Molecular Research Center, Cincinnati, OH) following the manufacturer’s protocol. RNA was reverse transcribed to cDNA with MMLV reverse transcriptase (Promega, Madison, WI). Real-time quantitative PCR was conducted using a LightCycler 480 instrument on cDNA using primer sets described in **Table 2** with LightCyler 480 SYBR Green I Master Mix (Roche, Indianapolis, IN). Results were normalized to 18S and are expressed utilizing the comparative Ct method (2^-ΔΔCt^) (28).

**Table 2:** Primers used for qRT-PCR.

Genes	Primer Sequence
**PYGL**	Forward: 5’-CAGCCTATGGATACGGCATTC -3’ Reverse: 5’-CGGTGTTGGTGTGTTCTACTTT -3’
**GAA**	Forward: 5’-TGCCCTCGCAGTATATCACAG -3’ Reverse: 5’-GAGACCCGTAGAGGTTCGC -3’
**18S**	Forward: 5’-ATCCCTGAGAAGTTCCAGCA -3’ Reverse: 5’-CCTCTTGGTGAGGTCGATGT -3’

### 2.8 Transfection

Transfections were performed using the DharmaFECT™ Transfection Reagents—siRNA transfection protocol (Horizon Discovery, UK) per the manufacturer’s instructions. Briefly, siRNA and DharmaFECT transfection reagent (Horizon Discovery, UK) were diluted with serum-free media. After 5 min incubation at room temperature, siRNA and transfection reagent were combined and incubated at room temperature for an additional 20 min. Antibiotic-free complete medium was added, media removed from samples and replaced with siRNA containing media for 48 hrs, and mRNA abundance assessed (qRT-PCR) to confirm gene depletion.

### 2.9 Transwell migration assay

Following transfection, cells were suspended in serum-free media and re-plated at equal densities into 8 μM transwells placed in a 24-well plate (28). Serum-containing media was placed in the bottom of the 24-well plate wells. Cells were incubated for 15 hrs at 37°C. Non-migrated and migrated cells were fixed to the transwells with methanol and stained using crystal violet in ethanol for quantification. Five random areas of the transwell were imaged and cell counts were averaged. Data are expressed as the percent of migrated cells.

### 2.10 ECM detachment assay

Extracellular matrix (ECM) detachment was simulated using Poly(2-hydroxyethyl methacrylate) (Poly-HEMA, Sigma) coated plates. Plates were coated with 70 µL/cm^2^ of 20 mg/mL Poly-HEMA in 95% ethanol. The plates were dried overnight under ultraviolet light after the addition of Poly-HEMA. The Poly-HEMA coated plates and dishes were rinsed twice with sterile calcium/magnesium-free phosphate-buffered saline before use. After cells were plated into the Poly-HEMA coated plates for 24 hrs, viability assays were performed according to the manufacturer’s instructions using 3-(4,5-dimethylthiazol-2-yl)-2,5-diphenyltetrazolium bromide (MTT, Sigma). Briefly, cells were plated into Poly-HEMA coated 96-well plates (20,000 cells/well). MTT solution was added to each well to a final concentration of 1% for 2 hrs, followed by solubilization in dimethylsulfoxide. Absorbance was measured at 570 nm and viability is expressed as relative absorbance readings.

### 2.11 Statistical analysis

Results are expressed as mean ± standard error of the mean. A two-tailed Students t-test or analysis of variance (ANOVA) tests were utilized to determine significance between two groups or across multiple groups, respectively. Statistical significance is considered P < 0.05.

## 3 Results

### 3.1 MCF10CA1a breast cancer cells have greater glycogen accumulation than MCF10A-*ras* cells and undergo rapid glycogen turnover

**Figure 1:**
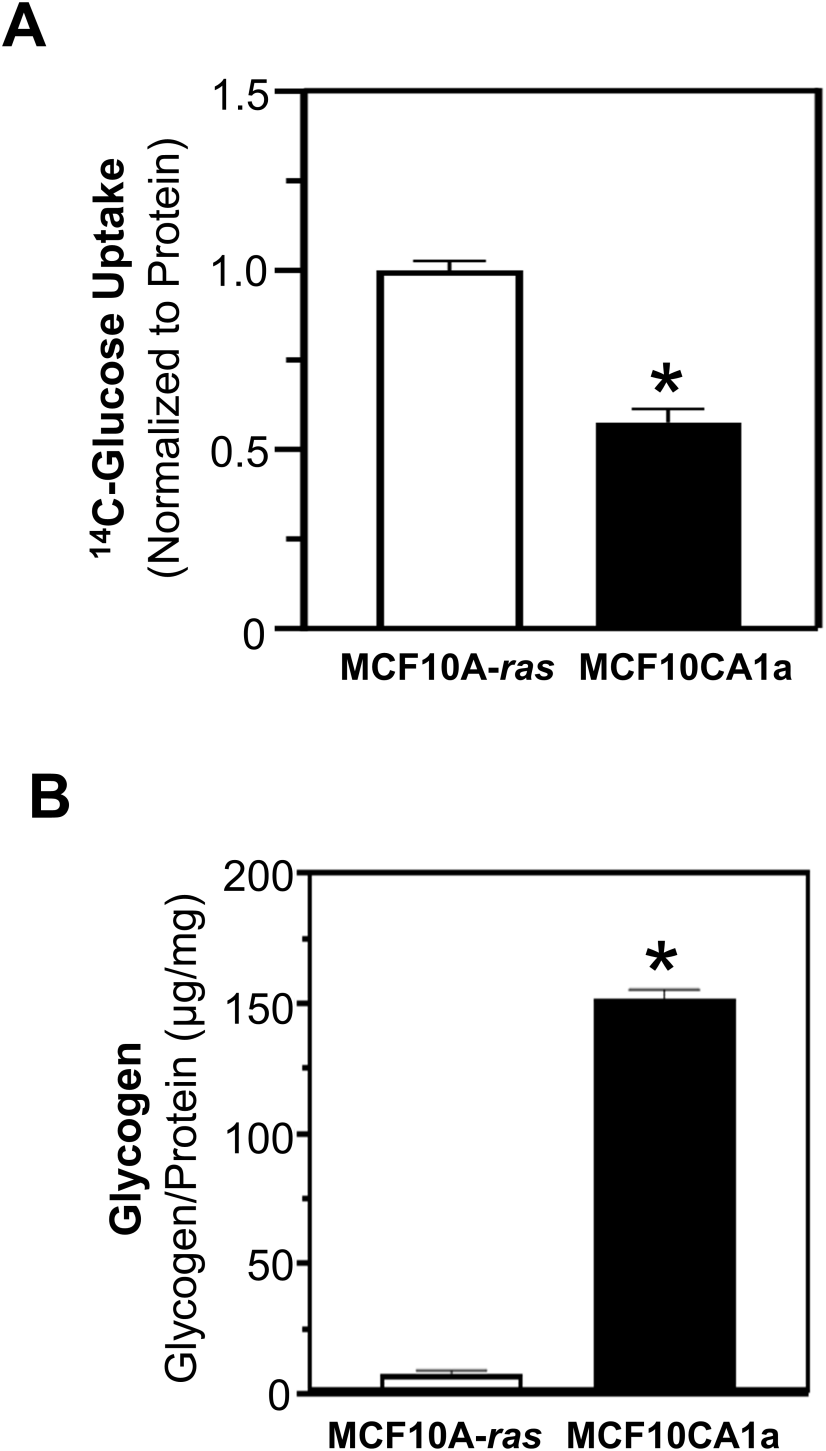
Glucose uptake and glycogen quantification of MCF10A-*ras* and MCF10CA1a. **(A)** Glucose uptake was assessed using ^14^C-Glucose incubated for 10 mins. Results are dpm/µg protein, normalized to MCF10A-*ras*. **(B)** Quantification of glycogen in MCF10A-*ras* and MCF10CA1a cell lines was assessed by glucose (GO) assay kit following the isolation of glycogen. Results are expressed as mean ± SEM. Asterisk (*) indicates significant difference compared to MCF10A-*ras* cells at P < 0.05 (n=4, representative of 3 experiments).

Glucose uptake and glycogen content in human metastatic MCF10CA1a breast cancer cells were compared to their nonmetastatic counterpart, MCF10A-*ras* cells. The glucose uptake was significantly higher in MCF10A-*ras* cells compared to MCF10CA1a cells (**Figure 1A**). However, the glycogen level in the MCF10CA1a was 20-fold greater than the non-metastatic MCF10A-*ras* (**Figure 1B**).

**Figure 2:**
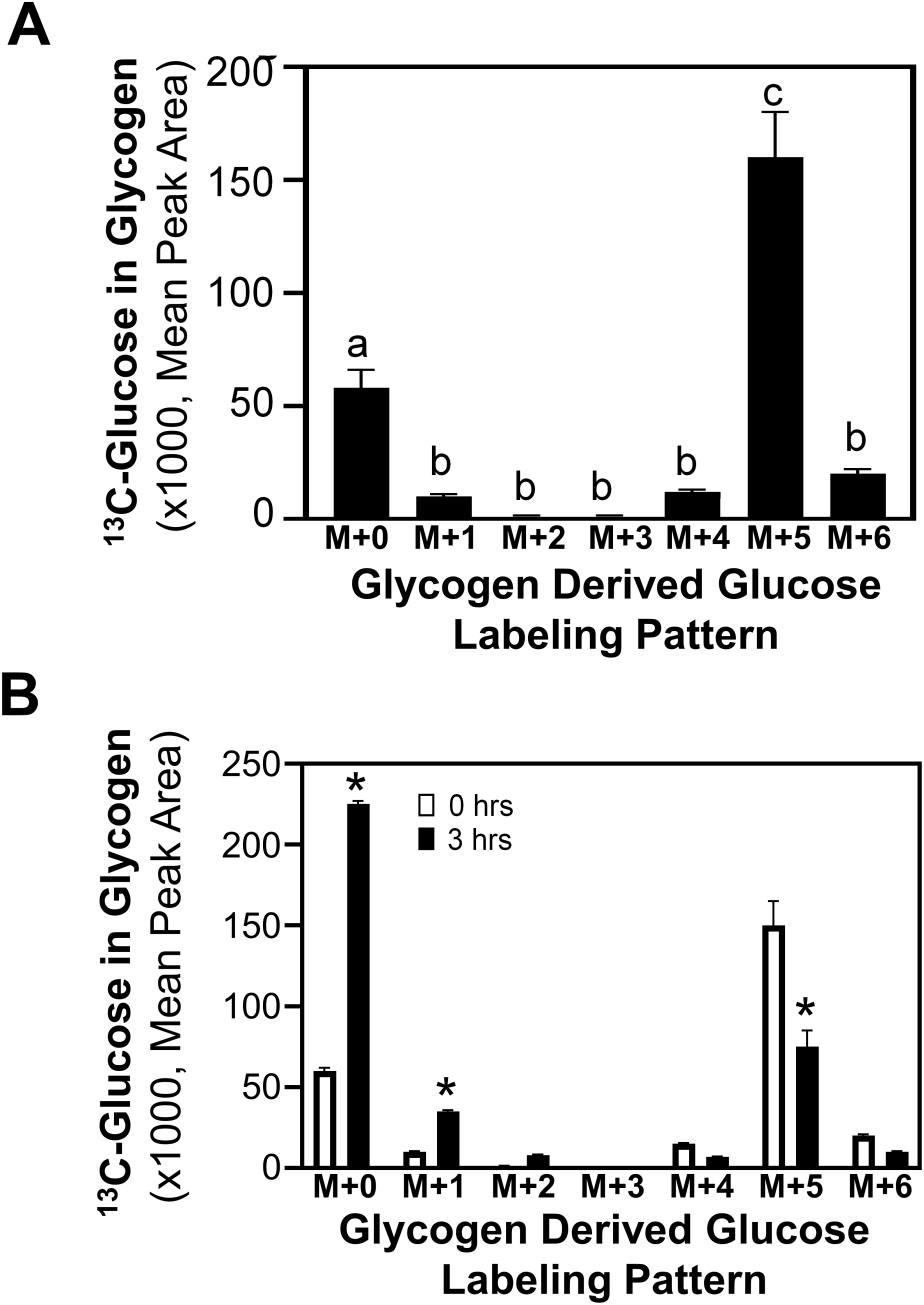
Labeled glucose distribution in glycogen. **(A)** ^13^C_6_-Glucose was utilized to determine glycogen synthesis and loss in MCF10CA1a cells through the labeling patterns of glucose stored in glycogen after 24-hr incubation with labeled media (n=4). The M+ indicates the number of labeled carbons in glycogen-derived glucose molecule. Statistical differences were assessed with ANOVA and bars with different letters indicate significant differences (P < 0.05, n=4, representative of 3 experiments). **(B)** ^13^C_6_-Glucose was utilized to determine loss of labeled glucose from glycogen of MCF10CA1a cells after 24 hrs incubation (0 hrs) followed by incubation with unlabeled media for 3 hrs (n=4). Asterisk (*) indicates significant difference between 0 and 3 hrs within each labeling group (P < 0.05, n=4, representative of 2 experiments).

To determine the pathway through which glycogen accumulates in metastatic MCF10CA1a cells, the incorporation of ^13^C_6_-glucose into glycogen was assessed. Following labeling cells with ^13^C_6_-glucose for 24 hrs, the form of glucose in glycogen was either unlabeled or primarily five ^13^C-carbon labeled glucose (M+5) (**Figure 2A**). Interestingly, ~2-fold greater glucose accumulation in glycogen was in the M+5 configuration compared to the unlabeled glucose. The M+5 labeling pattern suggests the potential of incorporation of glucose into glycogen through pathways other than the sequential phosphorylation upon entry into the cell, followed by the action of GYS to glycogenesis (15, 29). Glycolytic metabolism of ^13^C_6_-glucose produces ^13^C_3_ pyruvate, which is subsequently converted to oxaloacetate by the activity of pyruvate carboxylase (PC). Alternatively, the activity of pyruvate dehydrogenase (PDH) converts pyruvate to ^13^C_2_-acetyl CoA and, either through forward or reverse flux in the TCA cycle, produces oxaloacetate with 2 of the 4 carbons ^13^C labeled. Through these pathways, PCK can produce PEP with either 2 or 3 carbons labeled, which can produce M+5 labeled glucose that can be incorporated into glycogen. Alternatively, the labeled glucose may be metabolized through pentose phosphate pathway (PPP) to generate a 2 ^13^C labeled septoheptulose that can also be utilized in combination with 3-carbon labeled glyceraldehyde 3-phosphate (generated through the activity of PCK) to produce a 5-carbon labeled fructose 6-phosphate and subsequently an M+5 glucose metabolite. Further, to explore glycogen turnover, glycogen release was measured by labeling glycogen with ^13^C_6_-glucose for 24 hrs, removing the label, and assessing the subsequent reduction in ^13^C labeled glucose from glycogen. Interestingly, 3 hrs after removal of ^13^C_6_-glucose, levels of labeled glucose (M+5) stored in glycogen was reduced by approximately 50%, and unlabeled glucose in glycogen increased significantly (**Figure 2B**).

### 3.2 Gluconeogenic and pentose phosphate pathways mediate glucose incorporation into glycogen in metastatic MCF10CA1a cells

**Figure 3:**
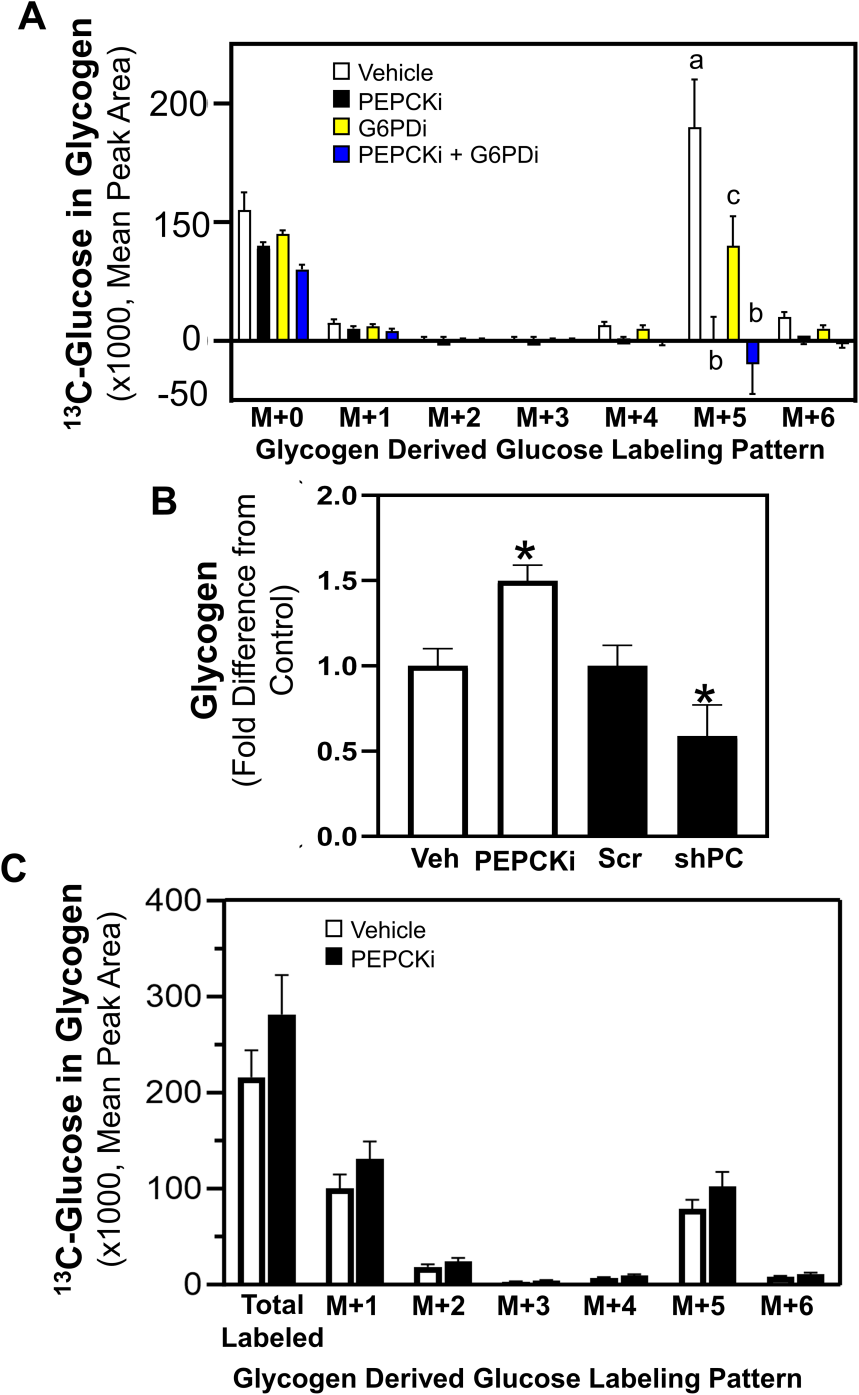
PCK and G6PD mediated glucose incorporation into glycogen. Shown in **(A)** ^13^C_6_-Glucose was utilized to determine glycogen synthesis of MCF10CA1a cells through the labeling patterns of glucose stored in glycogen after 24 hrs incubation with labeled media (n=4) with vehicle, PEPCKi, G6PDi or the combination of the inhibitors. The M+ indicates the number of labeled carbons in glycogen-derived glucose molecule. Bars with different letters indicate significant differences within the M+5 groups (P < 0.05, n=4). Results are representative of at least 2 experiments. **(B)** Cells were treated with PEPCKi for 24 hrs or 0.5 μg/mL of doxycycline for 3 days to induce PC depletion in shPC MCF10CA1a cells and glycogen accumulation assessed. Bars with asterisk indicate significant differences from vehicle and scram control, respectively (P < 0.05, n=4). Results are representative of at least 2 experiments. **(C)** Cells were incubated with ^13^C_6_-glucose for 24 hrs, washed and media replaced with vehicle or PEPCKi without labeled glucose. Labeling patterns of stored glycogen were determined after 24 hrs incubation (n=4).

In order to determine how glucose is metabolized from the media to achieve the identified labeling pattern of M+5 glucose stored in glycogen, pathways distinct from the direct incorporation of glucose from the media into glycogenesis were investigated. In addition to glycogenesis, glucose taken up from the media may enter the glycolytic pathway followed by gluconeogenesis, or enter the PPP [10]. A rate-limiting step in the gluconeogenic pathway is PCK, which converts oxaloacetate to PEP, ultimately forming glucose. In addition, glucose fluxes into the PPP through glucose-6-phosphate dehydrogenase (G6PD) and connects to the glycolytic pathway through the production of glyceraldehyde-3-phosphate to potentially produce a 5 carbon ^13^C-labeled fructose 6-phosphate, which can be incorporated into glycogen. Chemical inhibitors were utilized to inhibit the activity of cytosolic PCK (PEPCKi) and G6PD (G6PDi), and universally labeled ^13^C_6_-glucose incorporation into glycogen was determined. Inhibition of PCK eliminated the M+5 labeled glucose significantly compared to vehicle control (**Figure 3A**). Further, G6PD inhibition resulted in a partial reduction of the M+5 label in the glucose stored in glycogen, and a combination of both PCK and G6PD inhibitors completely eliminated the M+5 glucose label in glycogen (**Figure 3A**). These results suggest that the M+5 glucose labeled glycogen is derived primarily through the activity of PCK1.

To further investigate the role of PCK in glycogen accumulation, the impact of PCK1 inhibition or reduction of PC expression, which converts pyruvate to oxaloacetate to provide substrate for PCK, were assessed. Notably, inhibition of PCK1 (PEPCKi) increased glycogen accumulation, whereas PC-depletion (shPC) reduced glycogen accumulation (**Figure 3B**). Further, cells were pre-labeled with ^13^C_6_-glucose for 24 hrs and media was replaced with vehicle or PEPCKi without labeled glucose for another 24 hrs. Inhibition of PCK1 did not alter the labeling pattern of the ^13^C_6_-glucose in glycogen (**Figure 3C**). These results suggest that the increased glycogen accumulation after PCK1 inhibition is not due to inhibition of glycogenolysis. Instead, it is plausible that the increased accumulation of TCA cycle intermediates may increase synthesis via a path alternative to gluconeogenesis, mediated by PCK1 or PCK2.

### 3.3 Glycogenolysis and glycophagy mediate migration and viability in extracellular matrix detachment in metastatic MCF10CA1a cells

**Figure 4:**
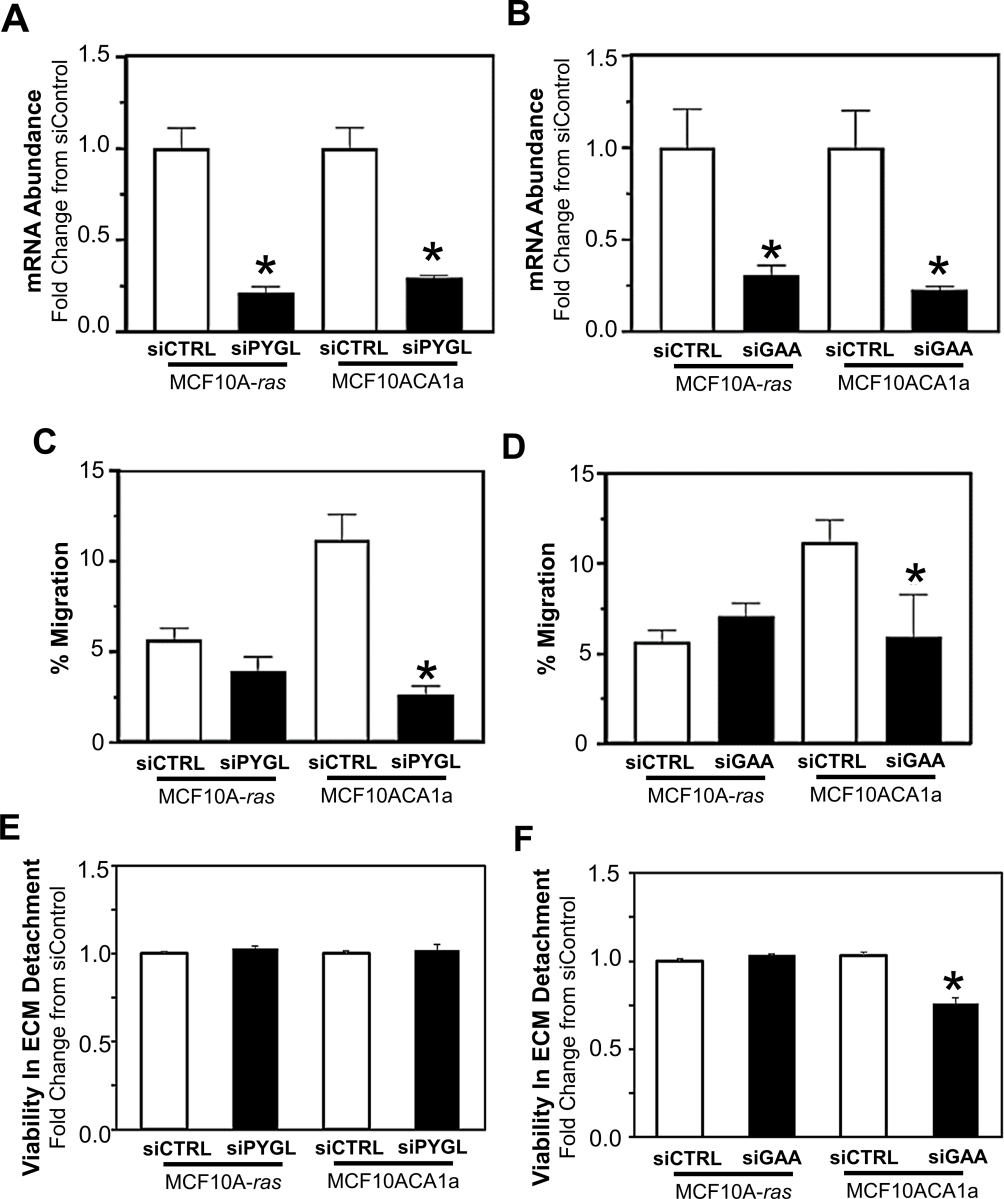
Effect of inhibition of glycogen release on cell migration and ECM detached viability of MCF10A-*ras* and MCF10CA1a cells. To examine the role of PYGL and GAA, **(A)** PYGL and **(B)** GAA expression was reduced employing siRNA in non-metastatic MCF10A-*ras* or metastatic MCF10CA1a. Migration of MCF10A-*ras* or MCF10CA1a cells following reduction in PYGL **(C)** or GAA **(D)**, which regulate glycogen degradation via glycogenolysis or glycophagy, respectively. Viability in ECM detached conditions with reduction in the expression of either PYGL **(E)** or GAA **(F)** in MCF10A-*ras* and MCF10CA1a cells. Results are expressed as mean ± SEM. Asterisk (*) indicates significant difference compared to siCTRL (P < 0.05, n=4, representative of 2 experiments).

Due to the rapid release of glucose from glycogen (**Figure 2B**), the role of PYGL and GAA in the migration and viability of ECM-detached MCF10A-*ras* and MCF10CA1a cells were determined. Following siRNA transfection, the inhibition of glycogenolysis (siPYGL) and glycophagy (siGAA) (**Figures 4A, B**) decreased migration of the metastatic MCF10CA1a cells, but not the nonmetastatic MCF10A-*ras* cells (**Figures 4C, D**), which contain low levels of glycogen (**Figure 1**). In addition, depletion of GAA (**Figure 4F**), but not depletion of PYGL (**Figure 4E**), reduced MCF10CA1a cell viability in ECM detached conditions, but did not impact MCF10A-*ras* cell viability. These results suggest that glucose release from glycogen may support metastatic breast cancer behavior, including migration and ECM detachment survival, through both PYGL and GAA activity.

## 4 Discussion

**Figure 5:**
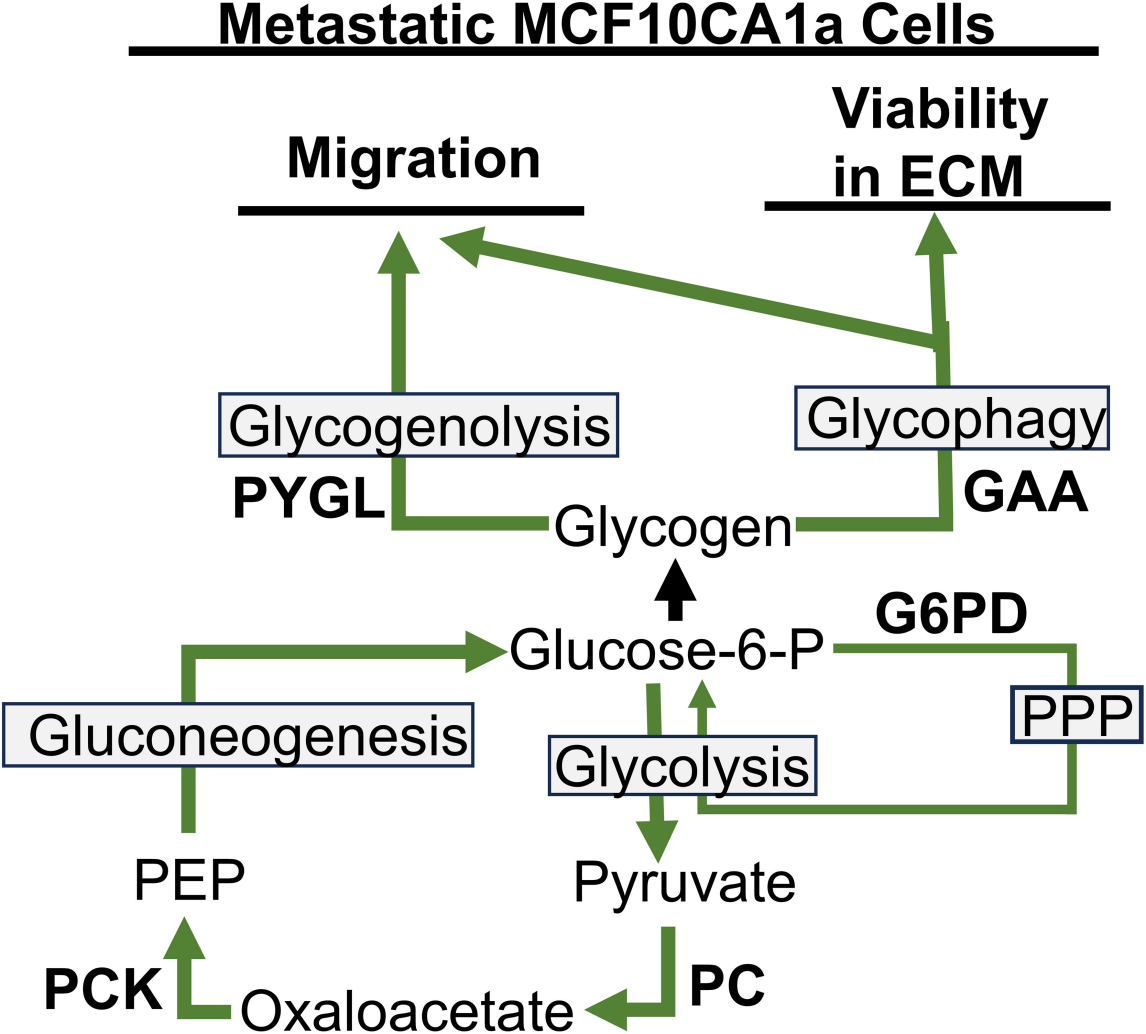
Pathways to glycogen accumulation and utilization for migration and viability in ECM detached conditions in metastatic MCFCA101a cells. PYGL, liver glycogen phosphorylase; GAA, lysosomal enzyme α-acid glucosidase; G6PD, glucose-6-phosphate dehydrogenase; PPP, pentose phosphate pathway; PEP, phosphoenolpyruvate; PCK, phosphoenolpyruvate carboxykinase; PC, pyruvate carboxylase.

Previous literature suggests that glycogen accumulation and utilization may play a role in the survival of cancer cells during the adverse conditions of metastatic progression; however, the metabolism and utilization of glycogen in cancer are not well understood. Our results show that metastatic MCF10CA1a cells have higher accumulation of glycogen compared to their non-metastatic counterpart, MCF10A-*ras* human breast cells, similar to the phenotype observed in clinical cancer samples (30). Our results indicate that the glucose incorporated into glycogen is generated through the activity of PCK, a rate-limiting step in gluconeogenesis, with a smaller contribution through G6PD of the PPP pathway, instead of the direct entry of glucose into glycogen (**Figure 5**). Further, while metastatic MCF10CA1a cells take up less glucose compared to the MCF10A-*ras* cells, they may instead rely on gluconeogenesis and PPP to generate glucose for glycogen stores. Furthermore, the ^13^C-glucose that enters the cell is rapidly incorporated into glycogen and the label is also rapidly depleted from glycogen, suggesting a high turnover rate of the glucose stored in glycogen from extracellular sources in the metastatic MCF10CA1a cells. Consistent with carbon flux into the TCA cycle that can supply oxaloacetate to gluconeogenesis, inhibition of PC reduced glycogen accumulation. Nonetheless, inhibition of PCK enhanced glycogen accumulation, suggesting stimulation of glycogen synthesis through other carbon sources and not reduced glycogen degradation as the sum of the remaining labeled glycogen pool following removal of the ^13^C-glucose after 3 hrs is not different between vehicle and PEPCKi treatment. In addition, enzymes responsible for release of glucose from glycogen, PYGL or GAA (via glycogenolysis and glycophagy, respectively), were required for migration, while only GAA was required for ECM-detached viability (**Figure 5**). Overall, our results suggest an alternative mechanism for the accumulation of glycogen through both gluconeogenesis as well as the PPP, and that accumulated glycogen within the MCF10CA1a cells is required for steps critical to metastasis. Thus, rapid turnover of glycogen may serve as an energy reserve to support the progression of breast cancer.

Our results suggesting a critical role of glycogen accumulation in cancers are consistent with the current literature. For example, glycogen-rich clear-cell breast carcinoma is associated with aggressive metastasis (31). Our results corroborate these clinical observations, which demonstrate that metastatic breast cancer cells have higher glycogen accumulation compared to their non-metastatic counterparts. Further, our results demonstrate that glucose is rapidly released. An example of a cancer-associated regulator of glycogen is the oncogene *RAB25* gene, which up-regulates HIF-1α activity in an oxygen-independent manner (23), and HIF-1α and hypoxia are shown to enhance glycogen concentration (18, 32). Thus, glycogen accumulation as assessed in our model system is associated with cancer progression.

Accumulation of glucose within glycogen stores is determined by the balance of glycogen synthesis and degradation, and the dysregulation of these pathways to promote increased accumulation in cancer cells is not well understood. Glucose synthesis can support utilization of glucose, glycogen storage and synthesis of lipids and amino acids for the rapid growth of cancer cells (10–13). Our results establish that PCK is required for the majority of glucose incorporation into glycogen. PCK overexpression has been identified in some tumor types and may therefore be important in promoting cancer initiation or progression (11). Expression is also enhanced in stressful circumstances which occur during metastasis, including by the hypoxia-driven transcription factor HIF-1α. HIF-1α also increases the expression of genes that drive increased glycolysis in cancer cells, thus providing a potential mechanism to coordinate glycolysis and PCK as noted in our results. Our results also indicate that flux of glucose from the media into the TCA cycle is required for the majority of the glycogen accumulation, as shown by the suppression of PC, but that TCA cycle intermediates may upregulate glycogen accumulation, as suppression of PCK expression increases accumulation.

Our results demonstrate the requirement for glycogenolysis and glycophagy for metastatic breast cancer cells to migrate, and glycogphagy to survive ECM detachment. Further, PYG and other mediators of glycogen degradation are associated with poor prognosis and diverse malignant phenotypes in cancers (15, 25, 33–35). Consistent with this, evidence demonstrates the role of PYG, and thus glycogenolysis, as a driver of cancer cell proliferation, survival, and protection from hypoxia-induced free radicals (17, 24).

Although the role of PYG in cancer cell progression is described in the literature, there is a gap exploring the association between glycophagy and cancer progression. Glycophagy plays a dynamic role in regulating normal cellular energy metabolism (16). However, evidence supports a role for glycophagy in cancer as a mutation of the STBD1 which induces lysosomal glycogen accumulation in cancer cells (36). Interestingly, a description of a ‘glycogen-addicted’ signature in clear cell carcinomas includes overexpression of common mediators of the glycogenesis pathway (i.e. RAB25, GLUT1, protein kinase B (AKT), GSK3, and AMP-activated protein kinase) (15, 17, 23). Thus, another source of available glucose from glycogen may provide enhanced metabolic adaptability to the varying environmental stressors that cancer cells experience, including glucose or other nutrient-deprived environments.

In summary, our results show that metastatic MCF10CA1a cells have higher glycogen accumulation compared to their non-metastatic counterpart, the MCF10A-*ras* cells. Additionally, glucose is incorporated into glycogen through PCK activity, a rate-limiting step in gluconeogenesis, with additional contribution from G6PD in the PPP pathway, instead of the direct entry of glucose into glycogen. Furthermore, the glucose that enters the cell is rapidly incorporated into glycogen and rapidly depleted, suggesting a high glycogen turnover rate in the metastatic MCF10CA1a cells. The enzymes responsible for glucose release from glycogen, PYGL or GAA via glycogenolysis and glycophagy, respectively, were determined to play a role in mediating migration as well as ECM detached cell viability. Collectively, our findings suggest that gluconeogenesis contributes to the accumulation of glycogen, and that glycogen utilization is required for steps critical to metastasis in MCF10CA1a cells.

## Data Availability

The original contributions presented in the study are included in the article/supplementary material. Further inquiries can be directed to the corresponding author.
